# Development of a Monoclonal Antibody Against Duck IFN-γ Protein and the Application for Intracellular Cytokine Staining

**DOI:** 10.3390/ani15060815

**Published:** 2025-03-13

**Authors:** Yingyi Chen, Wei Song, Junqiang Chen, Chenyang Jin, Jiewei Lin, Ming Liao, Manman Dai

**Affiliations:** 1 National and Regional Joint Engineering Laboratory for Medicament of Zoonosis Prevention and Control, Guangdong Provincial Key Laboratory of Zoonosis Prevention and Control, College of Veterinary Medicine, South China Agricultural University, Guangzhou 510642, China; chenyingyi1017@163.com (Y.C.); 13547572954@163.com (W.S.); junqiangchen@stu.scau.edu.cn (J.C.); jinchenyang187363@163.com (C.J.); 13425901895@163.com (J.L.); 2 UK-China Centre of Excellence for Research on Avian Diseases, Guangzhou 510642, China

**Keywords:** duck, IFN-γ, monoclonal antibody, intracellular cytokine staining

## Abstract

Interferon-γ (IFN-γ), a member of the Type II IFN family, is a crucial cytokine in the immune system and serves as an important indicator of immune response. Intracellular cytokine staining (ICS) is a technique used to analyze the production of cytokines within individual cells, and it has a wide range of applications in the fields of immunological monitoring, vaccine trials, and the study of infectious diseases. This study aimed to prepare monoclonal antibodies against duck IFN-γ protein and to establish an ICS protocol for detecting the duck IFN-γ protein. The *duIFN-γ-His* or *duIFN-γ-Fc* gene was cloned into the pEE12.4 expression vector and expressed as a recombinant protein of size 20.2 KDa or 54.9 KDa in 293F cells. The purified recombinant proteins were inoculated into BALB/c mice to generate splenic lymphocytes capable of secreting anti-duIFN-γ antibodies, and hybridoma cells were obtained after fusion with SP2/0 cells. A new hybridoma cell line named 24H4, which stably secreted IgG3 κ subtype antibody against duck IFN-γ, was established. This monoclonal antibody (mAb) was identified by Western blot to recognize duck IFN-γ antibodies, and the indirect ELISA results showed that its ability to recognize IFN-γ protein reached 0.001 μg/mL. The established ICS method was used to stain PBMCs after Concanavalin A (ConA) stimulation, and duck IFN-γ protein was successfully detected by flow cytometry, indicating that the ICS method was successful. In this study, we provide a crucial tool for subsequent research on duck cellular immune responses by using the monoclonal antibody 24H4.

## Simple Summary

Interferon-γ (IFN-γ) is a crucial cytokine in the immune system and serves as an important indicator of immune response. However, there are no commercially available monoclonal antibodies against duck IFN-γ. Intracellular Cytokine Staining (ICS) is a technique used to analyze the production of cytokines within individual cells. This study developed a monoclonal antibody against duck IFN-γ protein and established an ICS method using this antibody. This study will provide a crucial tool for the research of duck cellular immunity.

## 1. Introduction

Interferon gamma (IFN-γ) belongs to the Type II IFN family and plays an important role in inhibiting viral replication and regulating immune responses, primarily produced by CD8^+^ cytotoxic T lymphocytes (CTLs), Type I CD4^+^ helper T cells, and natural killer (NK) cells [1]. As a crucial cytokine-mediating immune response, the expression level of endogenous IFN-γ can reflect the immune status of an organism and is of significant value in studying immune mechanisms and functions, evaluating the effectiveness of vaccine immunization, and diagnosing pathogen infections [2]. Therefore, the expression level of IFN-γ serves as a marker for CD4^+^ and CD8^+^ T cell activation.

Ducks are important economic animals and one of the sources of animal protein [3]. Currently, ducks are threatened by various bacteria and viruses, including *Riemerella anatipestifer*, duck viral hepatitis, and duck enteritis virus, among others [4,5,6]. These diseases can lead to duck mortality or a decline in production performance, causing economic losses to the poultry industry. Furthermore, ducks are natural hosts of avian influenza viruses (AIVs), which exhibit strong infectivity and adaptability in ducks and play a crucial role in their spread [7,8]. Therefore, research on duck anti-infection immunity is necessary. Currently, most immune research on ducks focuses on innate immunity, with less emphasis on adaptive immunity, partly due to the lack of reagents and technical method limitations [9]. The RT-qPCR method was used to detect the mRNA level of duck *IFN-γ*, but it could not detect the protein level of duck IFN-γ [10]. The use of the ELISA method compensated for this deficiency, but it was unable to localize individual cells [11]. The Elispot method could localize to individual cells, but it could not determine the cell type that expresses IFN-γ [12]. Establishing a method that can detect both cell types and IFN-γ expression would be meaningful for evaluating duck cellular immunity.

Intracellular cytokine staining (ICS) is a common method used to measure the expression of cytokines in immune cells, especially T cells, at the single-cell level [13]. It is widely used in the fields of human and mouse infection, verification, and cancer research [14,15,16]. In studies on chickens, the ICS method has been used for screening viral epitopes and evaluating vaccines [17]. Due to the lack of corresponding antibodies for ducks, this method has not been applied to detect cytokine expression in ducks.

In this research, we disclosed a method for preparing a duck IFN-γ recombinant protein and have prepared a mouse anti-duck IFN-γ monoclonal antibody, 24H4. The ICS method was successfully established to detect the IFN-γ protein expression level in duck T cells. This study developed a monoclonal antibody, 24H4, which provides a crucial tool for subsequent research on duck cellular immune responses.

## 2. Materials and Methods

### 2.1. Ethics Statement

All animal research projects were approved by the Experimental Animal Ethics Committee of South China Agricultural University (identification code: 2023f018, 20 February 2023). All animal procedures were performed under the regulations and guidelines established by this committee and international standards for animal welfare.

### 2.2. Plasmids and Cells

The *duIFN-γ* base sequence was optimized and synthesized by AbMax Biotechnology Co., Ltd. (Tianjin, China) based on the duck IFN-γ amino acid sequence (NCBI accession number: Q9YGB9) and was cloned into the pEE12.4 expression vector. The Fc-tag and six-His-tag were synthesized by AbMax Biotechnology Co., Ltd. (Tianjin, China). The human embryonic kidney cells (HEK293F) were maintained in 293-cell chemically defined high-density serum-free cell culture medium (Kairui biotech, Zhuhai, China) at 37 °C in 5% CO_2_. The SP2/0 cells were maintained in Dulbecco’s Modified Eagle Medium (DMEM) with 10% fetal bovine serum (FBS) at 37 °C in 5% CO_2_. H5N1 AIV infected duck memory PBMCs were cryopreserved in our laboratory [18].

### 2.3. Expression and Purification of the Recombinant IFN-γ Proteins

The pEE12.4-duIFN-γ-His plasmid and pEE12.4-IFN-γ-Fc plasmid were transferred to 293F cells, and the cell supernatant was collected by centrifugation seven days later. DuIFN-γ-His protein and DuIFN-γ-Fc protein were purified using a Ni column or protein A preloaded column (Smart-Lifescience, Changzhou, China), respectively. The synthesized proteins were confirmed by SDS-PAGE analysis with Coomassie brilliant blue staining.

### 2.4. Preparation and Purification of the Monoclonal Antibody

The purified duIFN-γ-His protein was mixed with complete Freund’s adjuvant (sigma) or AD11.15 adjuvant (AbMax Biotechnology) and was inoculated in 6-week-old specific-pathogen-free female BALB/c mice (Charles River, Beijing, China). Four booster inoculations were performed after the previous immunization. Twenty-four days after the fourth immunization, serum antibody titers of BALB/c mice were detected by an Indirect Enzyme-Linked Immunosorbent Assay (ELISA). The mice with higher antibody titers were selected for shock immunization.

Monoclonal hybridoma cells were prepared using classical mAb production techniques. Briefly, splenocytes were resuspended with myeloma SP2/0 cells. After centrifugation at 1500 rpm, we discarded the supernatant and mixed the cells gently and then slowly added 1 mL 50% PEG at 37 °C for 25 min. After centrifugation at 1200 rpm, we discarded the supernatant and then resuspended the cells in DMEM medium and incubated them at 37 °C in 5% CO_2_. After incubating 48 h, the cells were transferred to HAT medium. The antibody content of their supernatant was detected with an indirect ELISA. Purified duIFN-γ-Fc protein was used as the envelope antigen, and the positive serum and SP2/0 cell supernatant of the mice were inoculated as positive and negative controls, respectively. The supernatant from the positive monoclonal hybridoma cell was collected and was purified using a protein A + G column.

### 2.5. Western Blot Analysis

The duIFN-γ-Fc protein samples, harvested from 293F cell supernatant and purified in a protein A preloaded column, were resolved by sodium dodecyl sulfate polyacrylamide gel electrophoresis (SDS-PAGE), transferred to a polyvinylidene fluoride (PVDF) membrane (Yeasen, Shanghai, China), and further incubated with mouse anti-duck IFN-γ antibody. After probing with primary antibodies, the blots were incubated with secondary antibody HRP-Goat Anti-Mouse IgG (Abcam, Cambridge, UK). The membrane was treated with HRP chromogenic solution (Merckmillipore, Bedford, MA, USA) and imaged using an infrared imaging system (Li-CoR, Lincoln, UK).

### 2.6. Antibody Subclass Detection

Subclasses of the monoclonal antibodies obtained above were identified using the commercial Mouse Monoclonal Antibody Isotype Elisa Kit (BIO-RAD, Hercules, CA, USA).

### 2.7. Intracellular Cytokine Staining

Two-week-old healthy mallard ducks (Sheldrake) were purchased from a duck farm in Guangzhou and housed in negative-pressure isolators. PBMCs were isolated from heparinized blood samples of healthy ducks using the lymphocyte separation medium as previously described [18]. A total of 3 × 10^6^ cells were seeded into the 48-well plate and stimulated with 2 μg/mL Concanavalin A (ConA) (Sigma-Aldrich, St. Louis, MO, USA) or H5N1 AIV at 37 °C in 5% CO_2_ for 12 h in the presence of 10 μg/mL Brefeldin A (BFA).

The cells were harvested and stained first with Mouse Anti-Duck CD8α (GeneTex, Irvine, CA, USA) for 30 min at 4 °C and then washed. The cells were then stained with FITC-conjugated Goat Anti-Mouse IgG2b (SouthernBiotech, Birmingham, UK). Next, the cells were then fixed for 20 min (in the dark) at 4 °C and washed according to the manufacturer’s instructions (BD Bioscience; Franklin Lake, NJ, USA). After that, the cells were stained with mouse anti-duck IFN-γ for 30 min and then PE-conjugated Goat Anti-Mouse IgG3 (SouthernBiotech, Birmingham) for 30 min. After washing, the samples were analyzed by flow cytometry (CytoFLEX; Beckman Coulter, Brea, CA, USA). The data were analyzed with FlowJo software (V10, Tree star Inc., Ashland, OR, USA).

### 2.8. Statistical Analysis

Statistical comparisons were made using GraphPad Prism 8 (GraphPad Software, San Diego, CA, USA). The results were presented as mean ± SEM. The paired/unpaired *t*-test and one-way ANOVA were used for statistical comparison. Statistical significance is indicated as follows: * *p* < 0.05, ** *p* < 0.01, and *** *p* < 0.001.

## 3. Results

### 3.1. Expression and Purification of Recombinant Duck IFN-γ Protein

**Figure 1:**
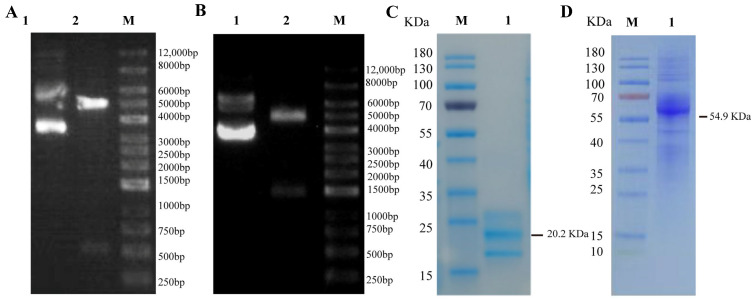
Expression of duIFN-γ-His and duIFN-γ-Fc proteins. *Eco*R I and *Hin*d III restriction enzymes were used to digest the duIFN-γ-His (**A**) and duIFN-γ-Fc (**B**) gene expression plasmids. Lane M: DNA ladder; Lane 1: duIFN-γ-His or duIFN-γ-Fc gene expression plasmid; Lane 2: production of digested duIFN-γ-His or duIFN-γ-Fc gene expression plasmid. SDS-PAGE analysis of purified duIFN-γ-His (**C**) and duIFN-γ-Fc (**D**). The duIFN-γ-His proteins were purified with Ni-NTA on a Ni-chelating column. The duIFN-γ-Fc proteins were purified with a protein A 4FF Chromatography Column. Lane M: protein molecular weight standard; Lane 1: supernatant of pEE12.4-IFN-γ-His (**C**) or pEE12.4-IFN-γ-Fc (**D**) vector expressed in 293F cells.

The duck *IFN-γ* gene, with His-tag or Fc-tag, was synthesized and then cloned into the pEE12.4 vector. The restriction enzyme digestion validation and sequencing results showed that the recombinant plasmids containing the genes of interest pEE12.4-IFN-γ-His (Figure 1A) and pEE12.4-IFN-γ-Fc (Figure 1B) had been successfully constructed.

The recombinant duck IFN-γ-His and IFN-γ-Fc proteins were successfully expressed through transfecting the pEE12.4-IFN-γ-His or pEE12.4-IFN-γ-Fc plasmids into the 293F cells. The supernatant from the transfected cells was collected seven days after transfection. The recombinant IFN-γ-His protein was purified with Ni-NTA on a Ni-chelating column. The recombinant IFN-γ-Fc protein was purified with a protein A 4FF Chromatography Column. The SDS-PAGE analysis showed that the band of interest obtained was 20.2 KDa (Figure 1C) or 54.9 KDa (Figure 1D), which is consistent with the expected size.

### 3.2. Preparation of the mAb Against Duck IFN-γ

**Figure 2:**
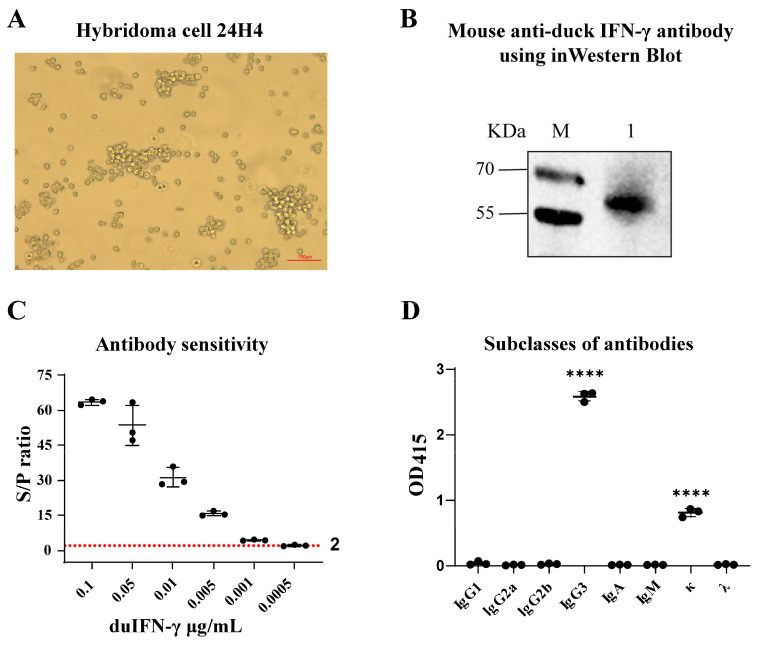
Characterization of mAb against duck IFN-γ protein. (**A**) Morphologic observation of 24H4 hybridoma cell. Scale bar, 100 μm. (**B**) Western blot identification of mAb. Lane M: protein molecular weight standard; Lane 1: Purified duIFN-γ-Fc protein. The purified duIFN-γ mAb was used as the primary antibody, and the goat anti-mouse IgG antibody was used as the secondary antibody. (**C**) IFN-γ antibody sensitivity was determined by indirect ELISA using duIFN-γ-Fc protein as the antigen and mAb 24H4 as the primary antibody. S/P ratio = sample OD_450_/NC OD_450_, S/P > 2 was considered positive. (**D**) Subclass of the mAb 24H4 was identified using a mouse antibody homotype ELISA kit; **** on the bar represents an extremely significant difference (*p* < 0.0001).

To obtain mAbs against duck IFN-γ protein, hybridoma cells were prepared with spleen lymphocytes from immune mice and SP2/0 cells, and a hybridoma cell that secreted mAb to anti-duck IFN-γ protein was screened and named 24H4 (Figure 2A).

After antibody purification, the binding ability of duck IFN-γ with mAb 24H4 was analyzed by Western blotting, and the results showed that recombinant duck IFN-γ-Fc protein could react with mAb 24H4 (Figure 2B). Using indirect ELISA to detect antibody sensitivity, it can identify duIFN-γ-Fc protein at a concentration of 0.001 μg/mL (Figure 2C). The subclasses of the secreting mAb were identified as IgG3 κ (Figure 2D).

### 3.3. Establishment an ICS Assay Protocol to Detect IFN-γ Expression in CD8^+^ T Cells from Duck PBMCs

To validate the absence of risk for antibody cross-reactivity in the established ICS protocol, mouse anti-duck CD8 was labeled with goat anti-mouse IgG3 subtype antibodies. The results demonstrated that the goat anti-mouse IgG3 subtype antibodies did not bind to the mouse anti-duck CD8 primary antibody, indicating that the ICS protocol we established carries no risk of antibody cross-reactivity (Supplementary Figure S1).

**Figure 3:**
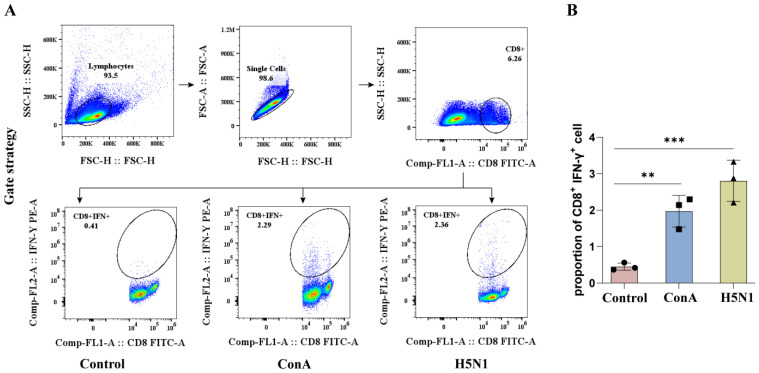
Flow cytometry analysis of IFN-γ expression in CD8^+^ T cells from duck PBMCs. (**A**) ICS gating strategy for the control group (duck PBMCs without any stimulation), the ConA group (duck PBMCs stimulated with ConA), and the H5N1 group (duck PBMCs from H5N1 AIV infected ducks). (**B**) Statistical analysis of IFN-γ expression in CD8^+^ T cells from duck PBMCs. The difference in IFN-γ expression between groups was assessed by one-way ANOVA and comparisons were considered significant at ** *p* < 0.01, *** *p* < 0.001.

The memory duck PBMCs were stimulated with ConA or H5N1 AIV and then subjected to ICS followed by flow cytometric analysis. Flow cytometric analysis was performed according to the gating strategy (Figure 3A). The results revealed that the prepared mouse anti-duck IFN-γ monoclonal antibody 24H4 could recognize IFN-γ produced by ConA or H5N1 stimulated duck PBMCs (Figure 3B), indicating that the prepared mouse anti-duck IFN-γ monoclonal antibody 24H4 is suitable for flow cytometric detection of changes in IFN-γ expression by duck immune cells.

## 4. Discussion

IFN-γ is an important immunomodulatory factor in ducks. Research on IFN-γ monoclonal antibodies for various livestock, including pigs, cattle, and horses, has been reported; however, to date, there have been no reports on monoclonal antibodies specific to duck IFN-γ [19,20,21]. In this study, we successfully expressed and purified the duck recombinant IFN-γ-His protein and the duck recombinant IFN-γ-Fc protein. Analysis of the amino acid sequence of the duIFN-γ protein (https://www.uniprot.org/uniprotkb/Q9YGB9/entry (accessed on 11 March 2025)) revealed the presence of three glycosylation sites (Asn42, Asn61, and Asn95). The purified duIFN-γ-His protein contained several additional bands, which may represent the glycosylated and non-glycosylated forms of duIFN-γ. A similar phenomenon has been observed in chIFN-γ expressed in the baculovirus system [22]. Additionally, one glycosylation site (Asn297) was located on the Fc-tag sequence. Although the theoretical molecular weight of the duIFN-γ-Fc protein was 54.9 KDa, the presence of various polysaccharide molecules, each with distinct molecular weights and abundances, resulted in a mixture of multiple glycosylated forms. Consequently, SDS-PAGE analysis revealed a diffuse band around 62.7 KDa, which was consistent with the results presented in Figure 2B. Moreover, we found that the IFN-γ-Fc protein showed diffuse bands between 35 and 40 kDa, likely due to incomplete expression of the Fc-tag, which still bound to the protein A purification column and was eluted. This portion of the protein was difficult to completely remove. Subsequently, using traditional hybridoma fusion methods, we established a hybridoma cell line (24H4) that secretes a monoclonal antibody against duck IFN-γ. This antibody, belonging to the IgG3 κ subtype, is capable of detecting IFN-γ protein at concentrations as low as 0.001 μg/mL. In addition, we found that the amino acid sequence similarity between chIFN-γ and duIFN-γ was 67%, and their three-dimensional structures were similar [23]. We investigated whether the mAb 24H4 could cross-react with chicken IFN-γ protein. The results showed that mAb 24H4 could detect chicken IFN-γ protein but did not recognize chicken IFN-α protein (Supplementary Figure S2).

IFN-γ is typically used as a marker of CD8^+^ T cell activation. CD8^+^ T cells recognize peptide-MHC class I complexes through the T cell receptor (TCR) and can proliferate and differentiate into specific cytotoxic lymphocytes (CTLs) [24]. CTLs exert their protective effects through a series of effector mechanisms, including the release of cytotoxic granules containing perforin and granzymes, inducing apoptosis through FAS/FAS-L interactions, and inducing the production of TNF-related apoptosis-inducing ligands and various pro-inflammatory cytokines [25,26]. In avian influenza virus (AIV) infections, several studies have clearly demonstrated the protective role of CD8^+^ T cells. For example, our previous studies confirmed that after mallard ducks were infected with the H5N1 subtype AIV, the proportion of CD8^+^ T cells, especially CD8^high+^ T cells, in duck PBMCs significantly increased. The Smart-Seq2 scRNA-seq results showed that H5N1 AIV infection mainly induces the signaling and functional activation of duck CD8^+^ T cells in vivo. These data indicated that the CD8^+^ T cells play a crucial role in combating H5N1 avian influenza virus infection [9]. Regrettably, due to the lack of duck IFN-γ antibodies, the study previously published did not employ the ICS method to investigate the specific CD8^+^ T cell immune response. The development of mouse anti-IFN-γ antibodies and the establishment of the ICS method in this study will remedy this deficiency and lay an important foundation for future research on duck T cell responses.

Screening for immunogens that can stimulate CD8^+^ T cell activation is of great significance for preventing and controlling viral infections such as AIVs. T cell epitopes refer to specific regions on antigen molecules that can be specifically recognized by the TCR on the surface of T cells and are believed to stimulate T cell responses [27]. In chickens, methods for screening T cell epitopes include IFN-γ ELISpot, ICS, and CFSE lymphocyte proliferation assays. In ducks, due to the lack of IFN-γ antibodies, techniques such as ELISpot and ICS cannot be effectively applied, and epitopes are mainly screened based on whether they can stimulate T cell proliferation and ELISA [28]. However, since the secretion of IFN-γ by CD8^+^ T cells cannot be detected, it is not possible to effectively determine whether peptides can stimulate CD8^+^ T cell activation. ConA is a commonly used non-specific T cell activator that can induce T cell activation and proliferation by binding to glycoproteins on the surface of T cells [29] In this study, we utilized ConA or H5N1 AIV to stimulate duck PBMCs and successfully detect IFN-γ production in CD8 T cells using the duck IFN-γ monoclonal antibody obtained in this research via ICS. The establishment of this ICS method provides a detection method for subsequent identification of duck-specific T cell epitopes.

In addition to identifying duck-specific T cell epitopes, the ICS method would play an important role in evaluating the cellular immune response to duck vaccines. In previous studies, the focus has often been on the evaluation of humoral immunity in ducks, while the study of cellular immunity has been neglected [30,31]. The development of duck IFN-γ antibodies and the establishment of the ICS method are of great significance for detecting cellular immunity in ducks. Duck IFN-γ antibodies form the basis for developing detection methods such as ELISpot and ELISA. These detection kits based on duck IFN-γ antibodies have greatly enriched the methods for evaluating duck vaccines, allowing for a more in-depth assessment of vaccine efficacy from multiple dimensions.

## 5. Conclusions

This study developed a mouse anti-duck IFN-γ antibody, 24H4. This antibody is of the IgG3 κ subtype and can recognize IFN-γ protein at a level as low as 0.001 μg/mL. This study also established an IFN-γ ICS staining method. This provides an important foundation for subsequent research on duck-specific T cell immune responses.

## Data Availability

The original contributions presented in this study are included in the article. Further inquiries can be directed to the corresponding authors.

## Supplementary Materials

The following supporting information can be downloaded at https://www.mdpi.com/article/10.3390/ani15060815/s1, Figure S1. Antibody cross-validation gating strategy. Figure S2. Validation of the specificity of mAb 24H4.
