# Supplementary figures and images for: Development of a Monoclonal Antibody Against Duck IFN-γ Protein and the Application for Intracellular Cytokine Staining
Source: Animals (Basel). 2025 Mar 13;15(6):815. doi: 10.3390/ani15060815 (PMC11939334; doi:10.3390/ani15060815)

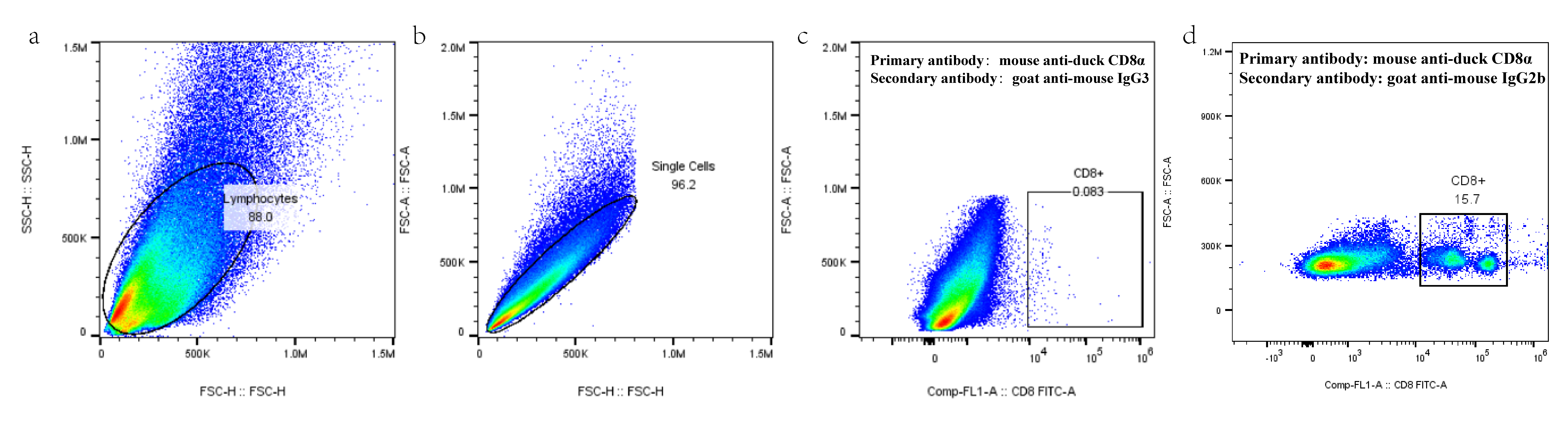

Supplement: Supplementary file 1 [file animals-15-00815-s001.zip › Supplementary Figure S1.tif]

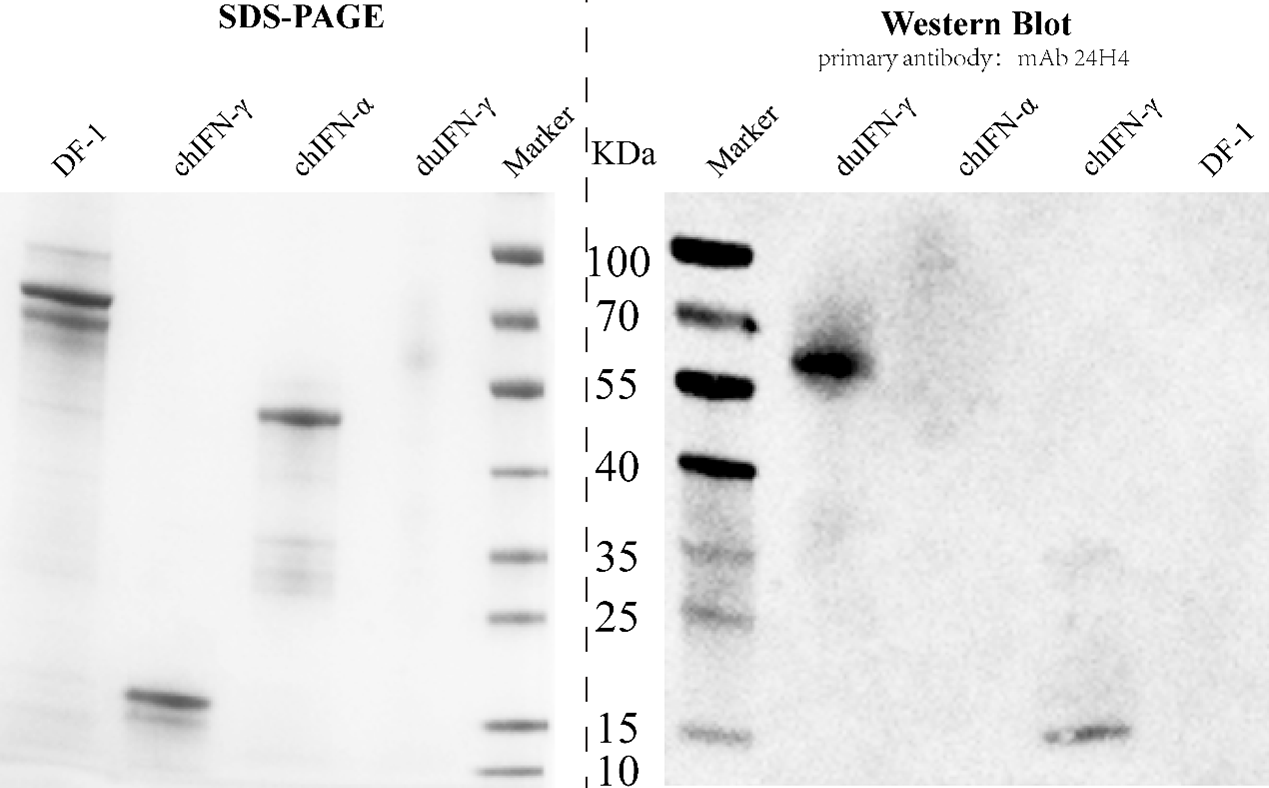

Supplement: Supplementary file 1 [file animals-15-00815-s001.zip › Supplementary Figure S2.tif]
